# Understanding How Heart Metabolic Derangement Shows Differential Stage Specificity for Heart Failure with Preserved and Reduced Ejection Fraction

**DOI:** 10.3390/biom12070969

**Published:** 2022-07-11

**Authors:** Federico Ferro, Renza Spelat, Camilla Valente, Paolo Contessotto

**Affiliations:** 1 Department of Medical, Surgery and Health Sciences, University of Trieste, 34125 Trieste, Italy; 2 Neurobiology Sector, International School for Advanced Studies (SISSA), 34136 Trieste, Italy; renza.spelat@libero.it; 3 Department of Molecular Medicine, University of Padova, 35122 Padova, Italy; camilla.valente@phd.unipd.it (C.V.); paolo.contessotto@unipd.it (P.C.)

**Keywords:** metabolomics, heart failure with reduced ejection fraction, heart failure with preserved ejection fraction, microbiota, biomarkers

## Abstract

Heart failure (HF) is a clinical condition defined by structural and functional abnormalities in the heart that gradually result in reduced cardiac output (HFrEF) and/or increased cardiac pressures at rest and under stress (HFpEF). The presence of asymptomatic individuals hampers HF identification, resulting in delays in recognizing patients until heart dysfunction is manifested, thus increasing the chance of poor prognosis. Given the recent advances in metabolomics, in this review we dissect the main alterations occurring in the metabolic pathways behind the decrease in cardiac function caused by HF. Indeed, relevant preclinical and clinical research has been conducted on the metabolite connections and differences between HFpEF and HFrEF. Despite these promising results, it is crucial to note that, in addition to identifying single markers and reliable threshold levels within the healthy population, the introduction of composite panels would strongly help in the identification of those individuals with an increased HF risk. That said, additional research in the field is required to overcome the current drawbacks and shed light on the pathophysiological changes that lead to HF. Finally, greater collaborative data sharing, as well as standardization of procedures and approaches, would enhance this research field to fulfil its potential.

## 1. Introduction

Heart failure (HF) is a pathological condition triggered by molecular and structural alterations that, although preserving physiological heart functionality, can act as a double-edged sword, finally leading to cardiac failure. Considering the enormous clinical and socioeconomical burden of HF, there is an urgent need to find improved diagnostic tools for both HF subtypes, “HFpEF” associated with preserved left ventricular ejection fraction (LVEF) and “HFrEF” with reduced LVEF. So far, the diagnosis of HF relies on symptoms’ assessment, physical examination reinforced by instrumental investigation, and the use of molecular biomarkers such as the natriuretic peptides and soluble suppression of tumorigenicity-2 and galectin-3. However, a growing body of evidence suggests that protein biomarkers are neither sufficiently sensitive nor specific and have limited diagnostic accuracy in clinical practice due to their variation in relation to age, obesity, gender [1], pulmonary, hepatic, and renal function [2,3]. The physiological mechanical flow of an adult heart is granted by constant adenosine triphosphate (ATP) generation from substrates such as fatty acids (FA) and glucose, as well as ketone bodies (KB), lactate, and amino acids (AA) via highly interconnected metabolic pathways, which include β-oxidation, glycolysis, the tricarboxylic acid cycle (TCA cycle), KB oxidation, and AA catabolism in cardiomyocytes mitochondria [4,5,6].

**Figure 1:**
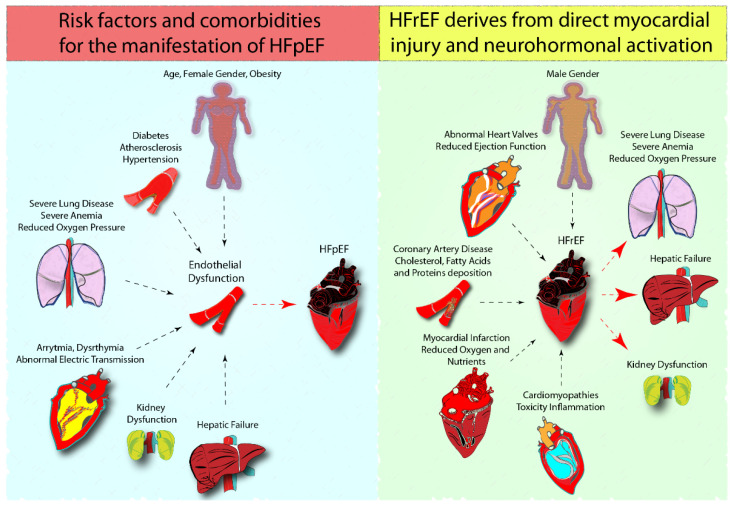
Correlation of HFpEF and HFrEF with microvascular dysfunction and direct myocardial injury, and neurohormonal activation. HFpEF is a complex clinical heterogeneous syndrome. An emerging paradigm emphasizes that HFpEF onset is favored by the combination of contributing risk factors (aging, female gender, obesity) and comorbidities (atrial fibrillation, hypertension, diabetes, kidney dysfunction, and chronic obstructive pulmonary disease), which cause endothelial dysfunction.

It is well-known that the clinical signs of HF are associated with a clear metabolic remodeling, which is made of alterations in metabolite absorption, trafficking, and utilization, finally culminating in decreased ATP production and, inevitably, cardiomyocytes contractility [4]. In addition, it is broadly acknowledged that aging, female sex, obesity atrial fibrillation, diabetes, and hypertension, together with other risk factors and systemic comorbidities, are primarily involved in the onset of HF with HFpEF as a result of endothelial dysfunction [7] (Figure 1). 

HFrEF is primarily caused by direct myocardial damage, coronary artery disease, myocardial infarction, cardiomyopathies, or inflammatory disorders and neurohormonal activation. All these pathological events induce hypoperfusion and poor venous drainage of visceral organs, thus leading to the development of co-morbidities, such as kidney, liver, and intestine failure.

In addition, HFrEF appears to be primarily driven by myocardial injury and subsequent neurohormonal activation with a male predominance [7,8] (Figure 1). 

Considering the well-known different etiologies and clinical symptoms between HFrEF and HFpEF, as well as the rising use of metabolomics as a reliable prognostic tool, in this review, we thoroughly evaluate the most recent clinical and preclinical evidence to explore the full potential of key metabolic alterations in the onset and progression of both HF subtypes.

### 1.1. Cardiac Metabolism of HFpEF and HFrEF and Preclinical Models

We acknowledge that HFrEF and HFpEF have distinct clinical manifestations in humans, and that animal models do not entirely replicate those features [9,10]. Although we recognize this limitation, we believe they might be effective in understanding more about the metabolic changes associated with HF. In reality, whereas HF slowly progresses in humans, HF-induced animal models minimize disease latency and may not result in the activation of the same molecular pathways, and hence biomarkers, along with bias due to reproducibility issues across various studies [11]. Another source of biological variability for HFpEF is the number of comorbidities in human illness, which is not considered in animal models. A further cause of discrepancy between animal models and human conditions is the unfortunate progression of HFpEF models into HFrEF, which is instead known to be a rare event in patients [12]. 

As a result, for the sake of simplicity, and because preclinical studies on cardiac metabolism rely on animal models that merely “mimic” clinical settings, we will use the terms “HFpEF” for those conditions associated with preserved LVEF and “HFrEF” for those associated with reduced LVEF throughout this review.

Different animal models have been developed for improving our understanding of the different pathophysiological causes related to HF. HFrEF models are mainly obtained through surgical procedures, such as transverse aortic constriction, coronary or abdominal artery ligation or constriction, pulmonary artery banding, rapid ventricular pacing, or a combination thereof. Additionally, transgenic animals have also been used to activate the HFrEF phenotype [13]. 

In comparison to HFrEF, the number of animal models that mimic HFpEF is limited. They replicate the principal factors known to trigger the development of HFpEF, specifically ageing, diabetes mellitus (leptin-deficiency obese/obese, leptin receptor-deficiency diabetic/diabetic), and hypertension (Dahl salt-sensitive rats). Furthermore, HFpEF models can be obtained via neurohormonal activation (Angiotensin II-treatment), and surgical procedures finely tuned to avoid inducing a reduction in EF (compensated hypertrophy induced by transverse aortic constriction) [13,14]. 

### 1.2. Fatty Acids Uptake and Oxidation

Fatty acids (FA) are the predominant energy source of cardiac cells in physiological conditions, accounting for 50–70% of total energy consumption [4]. In the cytosol, short- and medium-chain fatty acyl CoA can diffuse through the membrane inside mitochondria, while long-chain fatty acyl-CoA are actively transported into the mitochondrial matrix via acyl-CoA substitution with carnitine, generating the long-chain acyl carnitines (LCAC). Inside mitochondria, FA undergo β-oxidation, generating acetyl CoA, nicotinamide adenine dinucleotide (NADH), and flavin adenine dinucleotide (FADH2) [4]. 

Many studies on animal models with HFrEF andHFpEF reveal a significant downregulation of genes involved in pathways connected to FA uptake, transport, and catabolism, with a marked trend in the HFrEF group [15,16,17,18,19,20,21,22,23,24,25]. Indeed, animal models with early HFrEF (four weeks) have higher levels of medium- (MCAC) and long-chain acyl carnitines (LCAC) when compared to controls and models with HFpEF (four weeks) [18]. Furthermore, other studies found that the expression and activity of the MCAC processing enzymes did not change prior to overt HF [17,22,23]. 

Consistent with these observations, a significant impairment in FA uptake and oxidation was demonstrated in advanced-stage HFpEF animal models (9 weeks) [16], but not in the early stage (four weeks) [15,19,20]. Interestingly, cardiac degeneration from compensated hypertrophy to heart failure HFpEF in animal models was accelerated (one week) by the FA uptake-induced deficiency [24,25]. 

Recent clinical data indicate that HFpEF hearts obtain more energy from FA oxidation (42%) than HFrEF (35%) [26]. Studies based on large populations suggest a direct correlation between LCAC, MCAC, and the severity of LV systolic dysfunction [27,28,29]. A recent study found a substantial increase in MCAC at six- and twelve-month follow-up in patients with overt HF compared to healthy controls, lending validity to a stage-specific impact of MCAC in HFrEF [30].

However, studies including smaller cohorts of patients also showed higher LCAC levels in the HFpEF group with respect to HFrEF [31,32], correlating with endothelial dysfunction [33,34], arrhythmogenesis [35], and diabetes [36]. 

**Figure 2:**
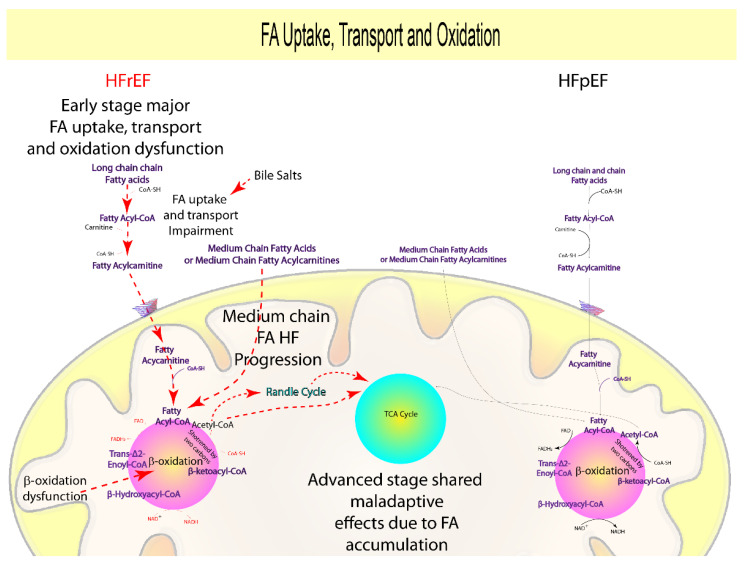
Suggested prognostic metabolites involved in FA metabolism. Increased plasmatic LCAC as a result of rapid FA metabolic impairment is an attractive indicator for diagnosing the early stages of HFrEF, as opposed to early-stage HFpEF, which has less impairment and a greater reliance. Furthermore, MCAC (i.e., octanoyl acid) may play a role in the progression of HFrEF from early to late stages, which is consistent with a neurohormonal and direct cardiac injury origin.

In summary, it could be assumed that an increase in plasmatic LCAC derived from an early FA metabolic impairment is a key signal to monitor the early stage of HFrEF as compared to early-stage HFpEF, where there is a smaller impairment and a larger dependence. Furthermore, MCAC may play a role in the progression of HFrEF from early to late stage, which is compatible with a neurohormonal and direct cardiac injury origin. Importantly, clinical data validated the preclinical findings that increased plasmatic LCAC and MCAC may be predictive of advanced HFrEF and HFpEF, consistent with a shared maladaptive effect induced by either neurohormonal and direct cardiac damage or microvascular dysfunction (Figure 2, Table 1). 

Importantly, clinical data confirmed preclinical results that elevated plasmatic LCAC and MCAC may be predictive of advanced HFrEF and HFpEF, consistent with mitochondrial oxidative stress being a common maladaptive effect in either neurohormonal and direct cardiac injury or microvascular dysfunction.

### 1.3. Ketone Bodies and Short-Chain Acyl Carnitines

Even though ketone bodies (acetoacetate, acetone, β-hydroxybutyrate (β-OHB)) and short-chain acyl carnitines (SCAC, acetate, butyrate, and propanonate) account for a minor portion of cardiac energy production, they are the most energy-efficient kinds of substrates available to cells [4,43]. KB and SCAC are produced by the gut microbiota or by FA breakdown in the liver; alternatively, KB are produced by protein catabolism [47]. Inside mitochondria, KB are oxidized by β-hydroxybutyrate dehydrogenase 1, succinyl CoA:3-oxoacid CoA transferase, and acetyl-CoA acetyltransferase 1, producing acetyl CoA and reducing equivalents, while SCAC are processed via β-oxidation machinery [4] or can be converted into FA and KB [110,115]. KB and SCAC metabolism plays a central role in the natural history of HF. Findings suggest that SCAC are preferred over ketones under physiological settings, and that this preference persists as heart failure progresses, as seen in both animal models and HF patients [43]. 

**Figure 3:**
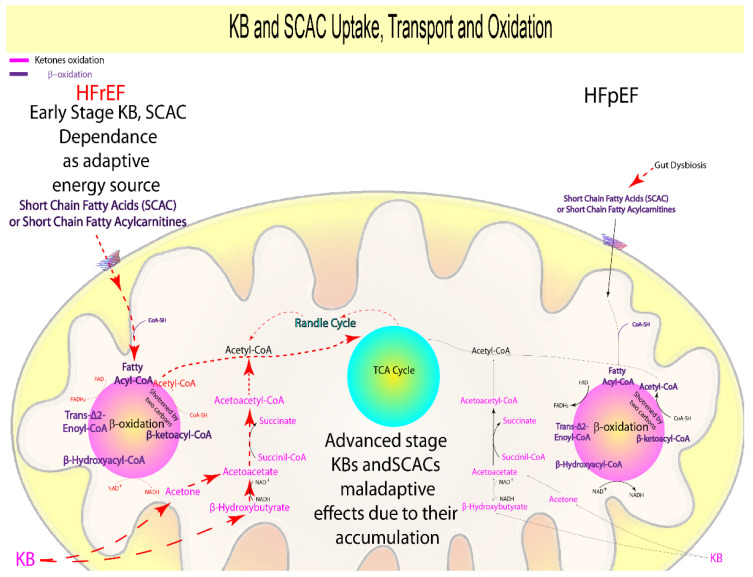
Suggested prognostic metabolites involved in KBs and SCACs metabolism.

However, KB enzymes and transporters’ expression and metabolic intermediates, such as hydroxybutyryl carnitine, acetyl carnitine, and succinate, are more abundant in animal models affected by early-stage HFrEF (four weeks) than in early-stage HFpEF models (four weeks) [44]. Further preclinical studies pointed out that KB are a more efficient energy source in animal models with early-stage HFrEF [45,46,51] (Figure 3, Table 1) than in HFpEF [52], and cardiomyocyte-specific suppression of succinyl CoA:3-oxoacid CoA transferase accelerates the progression from compensated hypertrophy to HFrEF in mice subjected to transverse aortic constriction [48]. 

KB, SCAC, and succinate consumption may be useful markers to identify HFrEF in the early stage as an adaptive energy source. Due to their maladaptive effect, KB and succinate accumulation in advanced stages makes them relevant as indicators of both HF subtypes, and congruent with clinical data supporting the neurohormonal and direct cardiac damage assumption and the disease’s microvascular dysfunction hypothesis. SCAC may also be important in advanced-stage HFrEF due to their maladaptive effect, which is coherent with the disease’s neurohormonal- and direct cardiac injury-derived origin (Figure 3, Table 1).

Supplementation with KB and SCAC reduces FA oxidation [43], and both show the ability to partly restore cardiac respiratory efficiency and proton driving force [43,45], as well as anti-inflammatory [43,50,52] and epigenetic effects [43,49,52]. 

Patients with HF have higher serum concentrations of KB and SCAC [43,53,54,57,58] as well as higher myocardial uptake and consumption in HFrEF patients compared to HFpEF patients [26]. Coherently, KB consumption in patients with HFrEF is three times higher than in HFpEF, and succinate release from cardiac tissues is slightly higher, but not significant, in HFrEF [26]. These findings suggest that higher KB and succinate levels are related to the worsening of the cardiac symptoms and ongoing local inflammatory conditions in patients with overt HFpEF and HFrEF [32,54,59].

Of note, increased KB and succinate among patients with HFrEF are associated with maladaptive effects, increased pro-brain natriuretic peptide [55], norepinephrine, growth hormone, and interleukin-6 secretion in the heart [56,60,63], while succinate accumulation was coupled with ischemia, mitochondrial reactive oxygen species (ROS) formation [62], and inflammation [64]. Consistent with previous observations, studies including patients with HF demonstrate that reduced SCAC plasmatic levels are inversely correlated with LV systolic function [30] and have or may have effects on pressure control [42,115,116]. 

Therefore, KB, SCAC and succinate consumption may be useful markers to identify HFrEF in the early stage as an adaptive energy source. Because of their maladaptive effect, KBs and succinate accumulation in advanced stage makes them relevant as indicators of both HF subtypes, and congruent with clinical data supporting the neurohormonal and direct cardiac damage assumption and the disease’s microvascular dysfunction hypothesis. SCAC may also be important in advanced-stage HFrEF due to their maladaptive effect, which is coherent with the disease’s neurohormonal and direct cardiac injury derived origin (Figure 3, Table 1).

### 1.4. Branched-Chain Amino Acids Oxidation

Branched-chain amino acids (BCAA, leucine, valine, isoleucine) are an energetic resource derived from protein catabolism for the whole human organism, including the heart. Specifically, BCAA can enter either into the TCA cycle (anaplerotic/cataplerotic reactions, valine, and isoleucine), or the KB oxidation path (ketogenic leucine, isoleucine), producing ATP, NADH, FADH2, and guanosine triphosphate [125,126]. A large body of literature demonstrates that BCAA, lipid, KB, and glucose oxidation are all closely related, and several pathways that can cross-regulate their metabolism have been proposed [5,6,71,127].

Several preclinical studies showed higher downregulation of the catabolic pathways associated with BCAA, and thus more accumulation of both BCAA and their catabolites in animals affected by HFrEF (both early- and advanced-stage) rather than in those with early-stage HFpEF [18,67,68]. Furthermore, a disease progression model from day one to four weeks [66] provided additional support in understanding the role of BCAA in HFrEF development. Therefore, it is clear that a marked impairment in BCAA metabolism begins in early-stage HFrEF and persists throughout the advanced phase [66], presumably contributing to hypertrophy, inflammation, vascular FA buildup, and altered insulin signaling [65,68,69,70].

Clinical data reveal that BCAA have a wide range of effects in HF patients [28,68,74,128,129]. Indeed, even though cardiac tissues from HFpEF patients absorb and likely consume more Ile and Leu than HFrEF patients [26], their buildup in overt HFpEF suggests a relationship with endothelial dysfunction [130]. Furthermore, it was proven that Leu and Ile oxidation is more dysregulated in HFrEF than in HFpEF patients [72]. In addition, patients with at-baseline higher LV dysfunction had higher plasmatic accumulation of valine, leucine [72], and Ile [72,73] than controls and patients with lower LV dysfunction.

**Figure 4:**
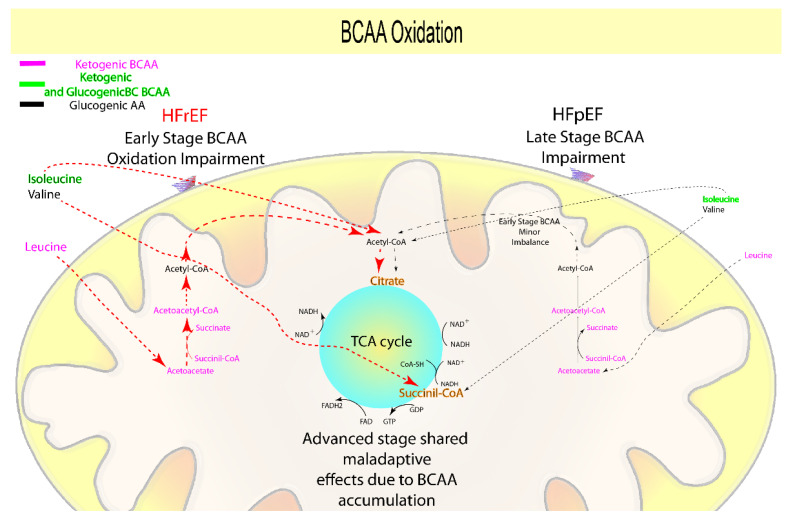
Suggested prognostic metabolites involved in BCAA metabolism: Accumulation of BCAAs (valine, leucine, isoleucine) or their catabolites may be suitable as predictors of early HFrEF, which is compatible with the neurohormonal and direct cardiac damage origins of the disease, as demonstrated by animal models. However, as the disease progresses, both HFpEF and HFrEF accumulate BCAA, causing maladaptive effects, which also supports the participation of the microvascular dysfunction in addition to the previously indicated neurohormonal-mediated and direct cardiac damage origin of the disease.

Consequently, the accumulation of BCAA or their catabolites may be suitable as predictors of early HFrEF, which is compatible with the neurohormonal-mediated and direct cardiac damage origin of the disease, as demonstrated in animal models. However, as the disease progresses, both HFpEF and HFrEF accumulate BCAA, causing maladaptive effects, and this also supports the dependence on the microvascular dysfunction along with the previously mentioned neurohormonal-mediated effect and direct cardiac muscle damage (Figure 4, Table 1).

### 1.5. Glycolysis and Glucose Oxidation

Glucose is the heart’s second most important energy source [4]: it crosses cell membranes via glucose transporters and enters the glycolytic pathway to create pyruvate, reducing equivalents, and ATP [131]. Pyruvate can be converted to lactate or transferred across the mitochondrial membrane by pyruvate translocase, allowing it to reach the TCA cycle [4].

The TCA cycle has a central role in metabolism consisting of different substrates and enzymes that work in a loop to produce ATP and reducing equivalents from the acetyl-CoA derived from glucose, FA, KBs, and AA [132]. TCA cycle activity is maintained by anaplerotic/cataplerotic reactions, in which metabolic substrates (such as AA) replenish TCA intermediates [125,126].

**Figure 5:**
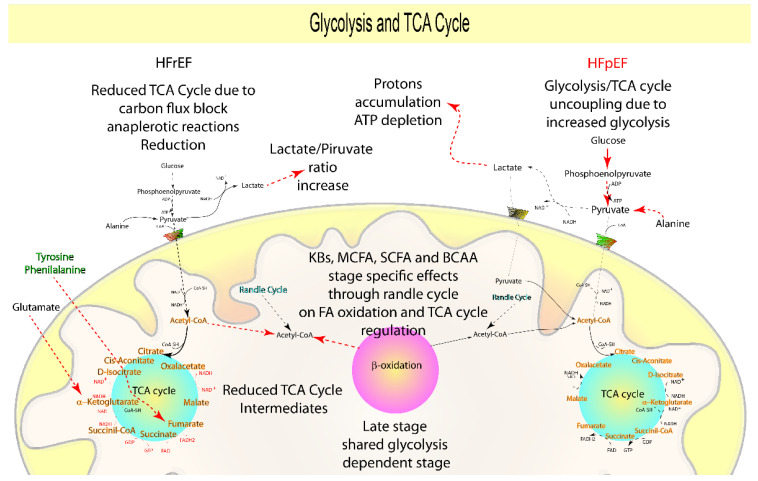
Suggested prognostic metabolites involved in glycolysis and TCA cycle metabolism: Even though clinical findings show that when HF progresses, whether in HFpEF or HFrEF, the heart compensates by switching to a glycolysis-dependent metabolism, indicating a direct and shared effect of neurohormonal and cardiac damage, as well as microvascular dysfunction, some differences have been discovered. To summarize, the rapid glycolysis-dependent progression of HFpEF caused by glycolysis-TCA uncoupling is emphasized by protons and glutamate build-up and Ala and lactate reduction, which may be used as biomarkers for early-stage HFpEF, thus reflecting its microvascular dysfunction origin. In contrast to HFpEF, glycolysis-dependent evolution in HFrEF occurs in a distinct manner, in accordance with a neurohormonal- and direct cardiac injury-derived origin. It begins in the early phase as a TCA carbon flux block (increased lactate), mainly derived from a reduction of the anaplerotic reactions accumulating glutamate, tyrosine, and phenylalanine, but it is probably slowed by the different metabolic conditions established during HFrEF onset.

When HF worsens, the heart changes to a glycolysis-dependent (fetal metabolism) phase to adapt, since glycolysis delivers an alternate source of ATP independent of oxidative metabolism to fulfill the heart’s metabolic requirements [4,47,51]. Nonetheless, animal studies show that distinct processes are implicated in the evolution of the glycolysis-dependent stage in HFpEF and HFrEF. Indeed, it was demonstrated that prior to the onset of diastolic dysfunction, HFpEF animal models (one to three weeks) show a rapid increase in glycolysis [15,16]. In the absence of FA oxidation dysfunction [15,16,19,20], the increase in glycolysis rate is followed by a reduced flux through the TCA cycle [19,20] with a null or weak reduction in lactate oxidation [15,20] (Figure 5, Table 1), resulting in their uncoupling and proton accumulation. As a result, proton accumulation favors pH reduction and lowers ATP stocks, used to remove protons and to maintain sodium and calcium homeostasis, resulting in decreased contractility [16,81].

Considering that insulin resistance, obesity, and diabetes are more frequent in HFpEF than in HFrEF patients, it is possible that these comorbidities may increase the glycolysis rate of the endothelial cells early in HFpEF, associating with vascular dysfunction [31,77].

Indeed, in animal models at early-stage HFrEF (one to three weeks), glycolysis remains stable or is reduced [75,76,78]. Furthermore, TCA intermediates, along with increasing lactate and the lactate/pyruvate ratio, begin to decline in the early stage (four weeks) [18] and continue to decline throughout the advanced stage (eight weeks) [17,85,86] of HFrEF. This was corroborated for HFrEF by showing stronger downregulation of genes involved in proline, alanine, and tryptophan catabolism and accumulation of phenylalanine, asparagine, aspartate, leucine, and Ile than in HFpEF animal models (four weeks) [18].

Previous results suggest a robust detrimental carbon flux block on the TCA cycle due to the reduction of the anaplerotic reactions in HFrEF animal models [17,18,24,25,75,76,78,85,86], consistently suggesting a delay in the glycolysis-dependent stage transition. The glycolysis-dependent progression in HFrEF may be delayed by the different conditions established during its onset (reduced FA and BCAA ox) in comparison to HFpEF, primarily via the Randle cycle [56,60,87,89] and as a result of the variable substrate (KB, MCAC, and SCAC) dependence during its progression [40,44,45,51,53,80,88,89].

As a result, the TCA carbon flow blockage in advanced HFrEF and the glycolysis-TCA uncoupling during the onset of HFpEF, both of which lead to ATP deficit and contractile dysfunction [16,40,44,53], may contribute to the clinically observed changes in lactate, alanine, glutamate, phenylalanine, and tyrosine (Figure 5, Table 1).

Lactate concentration is a prognostic factor for poor performance in HFrEF patients during the stress test [82], increased mortality in acute HF [63], myocardial infarction, and severe dilated cardiomyopathy, indicating a direct contribution to cardiac injury and HFrEF [83,84] (Figure 5, Table 1).

The plasmatic concentrations of glutamate [28,72,73], tyrosine [28,29,72,73], and phenylalanine [28,29,72,73,96,133] are lower in HFpEF, or individuals with lower LV dysfunction, and notably, other studies ascertained the opposite for Ala [31,54,72,92]. Alanine and glutamate are glucogenic AA, while phenylalanine and tyrosine are both glucogenic and ketogenic, and all of them are anaplerotic substrates that supply the TCA cycle [93].

Alanine is directly associated with an increase in glycolysis [90] and under pathological conditions with increased aerobic glycolysis/Warburg effect [91]. Furthermore, increased alanine is connected with microvascular disease, inflammation, and ROS production [31,74], and higher glutamate is instead related to cardiovascular disease and stroke [94].

Increased circulating phenylalanine levels have been linked to insulin resistance and greater protein catabolism [73,133]. Tyrosine in acute HF patients directly correlates with higher mortality at three months, via reduced synthesis of thyroid hormones, catecholamines, and neurotransmitters [63]. Notably, considering that a reduced Fischer’s ratio (the ratio of BCAA to aromatic amino acids phenylalanine and tyrosine) has been linked to hypoperfusion, impaired liver function, and HFrEF, it is reasonable to speculate that the rise in phenylalanine and tyrosine is related to such comorbidities [63,95].

As a result, while clinical data show that in overt HF, either HFpEF or HFrEF, the heart compensates by switching to a glycolysis-dependent metabolism, indicating a direct and shared effect of neurohormonal and cardiac damage as well as microvascular dysfunction, preclinical data reveal some differences.

In summary, the rapid glycolysis-dependent progression of HFpEF produced by glycolysis-TCA uncoupling is emphasized by protons and glutamate accumulation and alanine and lactate reduction, which may be utilized as indicators for early-stage HFpEF, thus reflecting its microvascular dysfunction origin.

In contrast to HFpEF, glycolysis-dependent evolution in HFrEF occurs in a distinct manner, in accordance with a neurohormonal- and direct cardiac injury-derived origin. It begins as a TCA carbon flux block (increased lactate) in the early phase, mostly generated from a decrease of the anaplerotic reactions accumulating glutamate, tyrosine, and phenylalanine, but is likely retarded by the different conditions established during its onset (Figure 5, Table 1).

### 1.6. Mitochondrial Dysfunction

**Figure 6:**
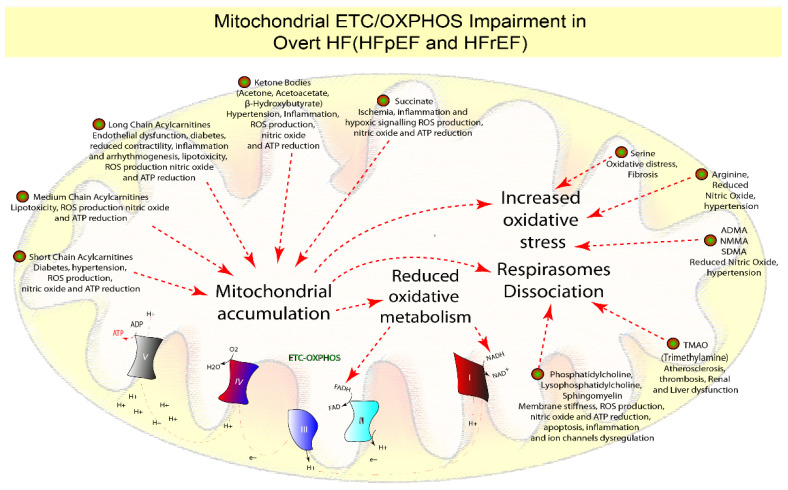
Suggested prognostic metabolites involved in ETC-OXPHOS dysfunction: Preclinical results suggest that mitochondrial dysfunction and decreased ETC-OXPHOS activity occur later after the beginning of both HF subtypes and are characterized by increased mitochondrial lipids, succinate, KB (mitochondrial dysfunction), asymmetric dimethylarginine (ADMA), symmetric dimethylarginine (SDMA), and N-monomethylarginine (NMMA) (oxidative stress), TMAO, or decreased phospholipids (respirasomes dissociation, complexes I, II, III, IV and V), arginine, and serine (oxidative stress). According to the microvascular dysfunction origin of the disease of HFpEF, decreased arginine and serine, as well as increased asymmetric dimethylarginine, symmetric dimethylarginine, and N-monomethylarginine, may be appropriate biomarkers at this stage, indicating mitochondrial dysfunction. Reduced phosphatidylcholine and an increase in TMAO in HFrEF may be useful indicators of mitochondrial dysfunction associated with neurohormonal dysfunction and direct cardiac damage disease-related origin.

The metabolic chain’s end purpose is ATP synthesis via oxidative phosphorylation (OXPHOS), whereby coupling oxidation to phosphorylation produces more than 95% of the ATP. During the process, highly energetic electrons from NADH and FADH2 are transferred through the five electron transport chain complexes (ETC) [4,134], across the inner mitochondrial membrane, to create an electrochemical gradient producing ATP, via ATP synthase (complex V) [4,134,135] (Figure 6, Table 1).

The phosphocreatine to ATP ratio decrease is a hallmark of both HF subtypes in overt conditions, indicating a deterioration in mitochondrial ETC-OXPHOS activity and ATP production [4,136].

It was revealed that ETC-OXPHOS and phosphocreatine to ATP ratio impairment develops in HFrEF animal models only 2–3 weeks after the procedure [78], and studies comparing early-stage HFrEF (four weeks) and HFpEF (four weeks) showed also modest impairment of genes associated to ETC-OXPHOS [18,44] and ATP synthase activity [15]. As a result, ETC-OXPHOS and ATP deficiency does not appear to be central during HF onset [15,18,44,78]. The ETC complexes are organized into respirasomes whose function is finely controlled according to the composition of the membranes [100,102]. Changes in the phospholipids’ composition result in respirasome dissociation [4,99,101], which reduces membrane fluidity and signal transmission [102,137], finally resulting in ETC-OXPHOS dysfunction, decreased ATP synthesis, and ROS generation [99]. Indeed, animal models with hereditary cardiomyopathy, that are characterized by a variable degree of LV dysfunction [97], have fewer phosphatidylcholine along with phosphatidylethanolamine and cardiolipin compared to wildtype animals [98].

As evidence, patients with HFpEF or HFrEF had lower levels of phosphatidylcholine [32,59,92], lysophosphatidylcholines [32,59,92], and sphingomyelins [32] than healthy individuals. In line with this, various authors suggest that increasing levels of trimethylamine N-oxide (TMAO), derived from phosphatidylcholine, choline, and carnitine in the liver, reflect a higher risk of atherosclerosis, thrombosis, and HFrEF [109,110,111], and it is also connected to patients with concomitant HFpEF and renal dysfunction, as evidenced by comparative investigations [108]. Numerous clinical studies on both HF subtypes have also shown that an imbalance in ROS generation, combined with increased mitochondrial accumulation of lipids [28,30,31,32,37,38,39,41], KB [26,32,53,54,55,56,57,58,60,61], and succinate [54,62,64], impairs OXPHOS/ETC activities. The final effect will be to induce mitochondrial damage via oxidative stress, inflammation, decreased nitric oxide, and ATP synthesis [4,79] (Figure 6, Table 1).

Moreover, while serine administration lowers cardiac fibrosis [103,104], its deficiency correlates with higher oxidative stress in both HFpEF [31,72] and HFrEF [73] (Figure 6, Table 1).

Lower levels of arginine are associated with higher levels of its catabolites (nitric oxide inhibitors), such as asymmetric dimethylarginine (ADMA), symmetric dimethylarginine (SDMA), and N-monomethylarginine (NMMA), which were found to be prognostic for HFpEF and endothelial dysfunction [31]. Additionally, a higher arginine reduction was demonstrated in a bigger cohort of HFrEF than in HFpEF [28], thus suggesting its relation with hypertension [105,106] (Figure 6, Table 1).

In conclusion, preclinical results suggest that mitochondrial dysfunction and decreased ETC-OXPHOS activity occur after the onset of both HF subtypes. In addition, they are characterized by increased lipids, succinate, KB, ADMA, NMMA, SDMA, TMAO, or decreased phospholipids, arginine, and serine. According to the microvascular dysfunction origin of the disease of HFpEF, decreased arginine and serine, as well as increased ADMA, NMMA, and SDMA, may be appropriate biomarkers at this stage, indicating mitochondrial dysfunction. Reduced phosphatidylcholine and an increase in TMAO in HFrEF may be useful indicators of mitochondrial dysfunction associated with neurohormonal-related origin and direct cardiac damage (Figure 6, Table 1).

### 1.7. The Microbiota Effect

Aside from the fact that HF is caused by intrinsic cell metabolic dysfunction, there is increasing evidence that nutrition, digestion, and microbiota are highly relevant. Indeed, the gut microbiota differs across genders and individuals, as well as in response to nutrition, resulting in variances that can either change nutrients’ absorption or produce metabolites with pathological effects.

Recently, an HFpEF animal model has been established by diet supplementation and mineralocorticoid treatment [107]. In addition, several authors suggest that levels of TMAO are associated with an increased risk of atherosclerosis, thrombosis, and kidney dysfunction [108,109,110], and with the risk of developing HFrEF [108,109,111]. However, it is still being discussed in the field that TMAO’s plasmatic negative effects are concentration-dependent (>10 μM), having either a positive (chaperone, osmolyte, and piezolyte) or a negative effect (atherosclerosis, thrombosis) [112]. It is also worth mentioning that TMAO is the metabolic byproduct of trimethylamine, which is produced either by the gut bacteria (exogenously) or endogenously, starting in either case from phosphatidylcholine, choline, and carnitine. Trimethylamine levels are associated with cardiovascular diseases [113,114] and potentially with HFrEF.

Moreover, the gut microbiota also produces short-chain fatty acids, the lack of which has been linked to hypertension, hypertrophy, fibrosis, and diabetes [42,110,115,116], and with HFpEF [117], thus suggesting their involvement in asymptomatic or early-stage disease.

In addition to their role in cholesterol/lipid absorption and glucose metabolism, bile acids have a direct influence on heart function and vascular tone via the farnesoid X receptor, the muscarinic M2 receptor [118,119], and the calcium-activated potassium channels [120]. Pathological effects are associated with increasing secondary/primary bile acids ratios [121], and both HF patients and HFrEF-induced animals (8 weeks) treated with primary bile salts showed an improved cardiac function through cardio-protection [122,123].

As a result, according to the disease’s neurohormonal and direct cardiac damage origin, increased trimethylamine synthesis and absorption may be a signal of early-stage HFrEF, and it may be important in overt HFrEF when combined with TMAO (Figure 6, Table 1). Due to their involvement in lipid adsorption and cardio-protection, primary and secondary bile acids ratio should be investigated in early-stage HFrEF (Figure 2, Table 1). Finally, while short-chain fatty acids are used as an adaptive energy source in early-stage HFrEF, their concentration in early-stage HFpEF should be assessed due to their relationship to comorbidities and gut dysbiosis, implying that the disease is caused by microvascular dysfunction (Figure 3, Table 1).

### 1.8. Metabolomic Fingerprinting and Clinical Perspective

**Figure 7:**
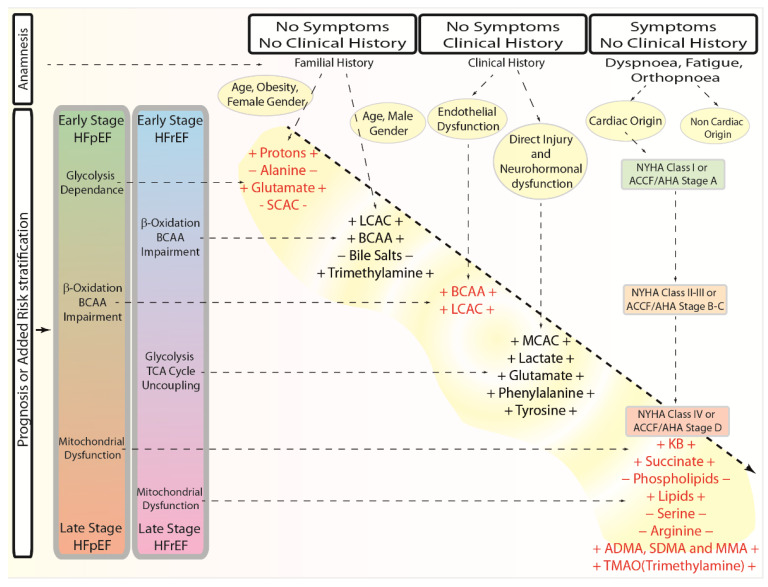
Flowchart of the proposed approach to consider metabolic markers’ expression in anamnesis, prognosis, or risk stratification of HFpEF and HFrEF.

A considerable amount of preclinical evidence is based on metabolomics, but correlative clinical findings are rare. Therefore, we believe that metabolomics has a huge potential that can only be achieved by testing and exploring its clinical application. In fact, metabolomics fingerprints of HF patients may enhance clinical experts’ prognostic and diagnostic methods in a variety of ways, whether in subclinical or clinical settings. To improve the current prognostic and diagnostic tools by integrating data from the metabolomics fingerprint, we propose a flowchart of a standard operating procedure (Figure 7).

In the absence of any signs, symptoms, or clinical history, a known familiarity of HF, combined with the patient’s anamnesis, would be the first evidence for a systematic and planned metabolic profile. Even though the technique’s versatility allows for the examination of either metabolites for HFpEF or HFrEF combined, the established traditional risk factors may indicate which subtype to analyze more specifically. In fact, according to the Framingham Study, HFpEF is two times as frequent in women [138] and more often associated with hypertension, obesity, and diabetes type 2 [139]. The fact that women are more likely to have HFpEF [7,31,139,140,141,142] is reflected in the evidence that low estrogen levels are associated with increased oxidative stress, inflammation, and endothelial dysfunction [143]. Men, on the other hand, are more susceptible than women to developing HFrEF as a result of direct damage and neurohormonal activation, whereas older men with chronic renal insufficiency are more likely to develop HFpEF [144].

In this scenario, early-stage markers for HFpEF (protons, alanine, glutamate, and short-chain fatty acids) should be investigated in women or older men with renal insufficiency as a first choice, while early-stage HFrEF metabolic markers (LCAC, BCAA, bile salts, and trimethylamine) should be first investigated in men.

In the presence of a clinical history and the existence of comorbidities associated to HF pathology, the decision would be guided by clinical data and anamnesis. As previously stated, the onset of HFpEF is related to atrial fibrillation, hypertension, diabetes, kidney dysfunction, and chronic obstructive pulmonary disease. Therefore, those comorbidities suggest a specific metabolic fingerprint directed toward the search of the subclinical HFpEF metabolic markers. Therefore, in a direct proportional manner to the timeframe, duration, and intensity of the comorbidities, monitoring the levels of metabolites associated with early-stage HFpEF, such as protons, alanine, glutamate, and short-chain fatty acids, and then for BCAA and LCAC, would be a possible strategy.

In contrast, individuals that experienced coronary artery disease, myocardial infarction, cardiomyopathies, or inflammatory disorders should be identified as potential subclinical HFrEF patients. Therefore, proportionally to the event timeframe and duration of the comorbidities, checking for the presence of metabolites such as LCAC, BCAA, bile salts, or trimethylamine, and then MCAC, lactate, glutamate, phenylalanine, and tyrosine, would be the first aim at this stage.

Patients presenting with no clinical history but with symptoms such as dyspnea, fatigue, or orthopnea require the identification of the underlying etiology. In this context, the medical history and the physical examination can rule out whether the symptoms have a cardiac or non-cardiac origin, and metabolomics might be extremely useful in diagnosing and treating subclinical situations of either HFpEF or HFrEF rather than overt HF. In addition, when dealing with people with no clinical history but evidencing symptoms, either the New York Heart Association Functional Classification (NYHA) or the guideline for the management of heart failure from the American Heart Association/American College of Cardiology (ACCF/AHA) may be useful. In fact, the NYHA and AHA classifications might be utilized to individuate the relative stage and thus perform a specific metabolic profiling. Given the existence of established risk factors, a NYHA class I or an ACCF/AHA stage A might be matched to an early stage of both HF subtypes and thus evaluated for early-stage indicators. Moreover, a NYHA class III or an ACCF/AHA stage C-D patient should be screened for early-stage as well as metabolic markers involving β-oxidation, BCAA impairment in HFpEF patients, or early-stage HFrEF markers, and metabolites involved in glycolysis TCA cycle uncoupling in HFrEF subclinical patients. Additionally, a NYHA class IV or an ACCF/AHA stage D case should be analyzed for late-stage metabolic indicators of mitochondrial failure (KB, succinate phospholipids, lipids, serine, arginine, ADMA, SDMA, MMA, and TMAO (trimethylamine)).

## 2. Conclusions

Here, we reviewed both preclinical and clinical studies showing distinctive metabolic profiles that evolved in relation to either the early or advanced stage of the different subtypes of HF. Namely, while HFpEF onset seems to rely on glycolysis (Ala, protons, lactate, and Glu), showing minor FA oxidation impairment (SCAC), early HFrEF clearly appears to be dependent on KB and SCAC oxidation, having greater FA adsorption (bile salts), transport, and oxidation (LCAC, MCAC), as well as BCAA catabolic deficiency. That said, it is plausible that the block of the carbon flux (phenylalanine, tyrosine, glutamate), together with the increased glycolysis-TCA uncoupling, in HFrEF, and the increasing FA and BCAA catabolic impairment in HFpEF, force both disease subtypes to progress from an early to an advanced stage. Finally, a marked alteration of phospholipids, TMAO (trimethylamine), LCAC, MCAC, SCAC, KB, succinate, serine, arginine, ADMA, NMMA, and SDMA leads to an increased ETC-OXPHOS impairment, mitochondrial oxidative stress, and inflammation in both HF subtypes.

Even though animal studies show substantial differences between the HF subtypes, clinical results reveal greater heterogeneity, echoing the large inter- and intra-individual variability, also dependent on dietary habits and microbiota, within each cohort.

However, the main reasons why we do not routinely use metabolomics in clinical settings are due to the presence of issues related to equipment settings, experimental validation, method standardization, and the interpretability of reliable and reproducible data [145]. Furthermore, as with other plasmatic or humoral indicators, effective threshold values within the healthy population are missing. Hopefully, by fostering collaboration among different research groups, simplifying data exchange, and comparing bigger datasets across different cohorts, it will be possible to boost the clinical applications of metabolomics. Clusters and societies are already blooming, aiming to both reduce the heterogeneity and variability of the metabolomics results and improve the predictive power of the data. In fact, Biocrates (https://biocrates.com, accessed on 1 July 2022), Metabolomics Society (http://metabolomicssociety.org, accessed on 16 June 2021), COnsortium of METabolomics Studies (COMETS) (https://epi.grants.cancer.gov/comets, accessed on 16 June 2021), and Phenome and Metabolome aNalysis (PhenoMeNal) (https://phenomenal-h2020.eu, accessed on 1 July 2022) are four of such groups. Furthermore, identifying and selecting stage- and subtype-specific metabolic panels would make a significant advance in the early diagnosis of individuals at higher risk of developing HF. Metabolomics, in this context, has the strong potential to become a key tool within the concept of P4 medicine: prevent, predict, personalize, and participate, also enabling the development of new metabolic modulators with therapeutic effects.

## Figures and Tables

**Table 1:** Summary of metabolic alterations and relative metabolites associated with HFrEF and HFpEF. Physiological and pathological effects are listed according to the relative metabolic markers.

Metabolite, Pathway	Early HFrEF	Adv HFrEF	Early HFpEF	Adv HFpEF	Physiological Effect	Pathological Effect	Ref.
*Fatty Acids*, *Acylcarnitines oxidation*	*− − −*	*− − −*	*=*	*− − −*	*Fatty Acids β-Oxidation*	*FA accumulation*	[15,16,17,18,19,20,21,22,23,24,25]
**Long-Chain Acylcarnitines Oxidation**	+	+	=	+	ATP production, ketone bodies formation, FA oxidation	Diabetes, reduced contractility, inflammation, arrhythmogenesis, lipotoxicity, ROS production, nitric oxide and ATP reduction	[26,27,28,29,31,32,33,34,35,36,37,38,39,40,41]
**Medium-Chain Acylcarnitines Oxidation**	+	+	=	+	ATP production, ketone bodies formation, FA oxidation	Transition to HF, lipotoxicity, ROS production, nitric oxide and ATP reduction	[28,30,32]
**Short-Chain Acylcarnitines Oxidation**	+	+	=	=/+	ATP production, ketone bodies formation, FA metabolism	Diabetes, hypertension, ROS production, nitric oxide and ATP reduction	[26,30,31,42,43]
*Ketone bodies oxidation*	*+*	*− − −*	*=*	*− − −*	*Ketone bodies oxidation*	*KBs accumulation*	[43,44,45,46,47,48,49,50,51,52]
**KBs Oxidation (Acetone, Acetoacetate, 3-Hydroxybutyrate)**	+	−	=	−	ATP production, anti-inflammatory, epigenomic regulation	Hypertension, Inflammation, ROS production, nitric oxide and ATP reduction	[26,32,43,45,46,47,49,50,51,52,53,54,55,56,57,58,59,60,61]
**Succinate**	+	−	=	−	TCA cycle intermediate, ketone bodies formation, FA oxidation	Ischemia, inflammation, and hypoxic signaling, ROS production, nitric oxide and ATP reduction	[37,54,55,56,60,62,63,64]
*Branched-Chain Amino Acids oxidation*	*−*	*− − −*	*=*	*− −*	*BCAA oxidation*	*BCAA accumulation*	[5,18,65,66,67,68,69,70,71]
**Leucine, Isoleucine, Valine**	+	+	=	+	Anaplerotic reactions, ketone and short-chain fatty acids oxidation	Pro-anti-hypertrophic and pro-anti-inflammatory, FA accumulation	[5,18,28,29,32,54,71,72,73,74]
*Glycolysis*	*=*	*+ + +*	*+*	*+ + +*	*Glucose anaerobic metabolism*	*Lactate and protons accumulation*	[15,16,19,20,24,25,46,47,75,76,77,78,79,80,81]
**Protons**	=	+	+	+	ATP production	Reduced contractility, troponin I calcium binding, calcium current generation, and ATP availability	[15,16,77,81]
**Lactate**	+	+	=	=/−	Glycolysis, Glucose Oxidation	Myocardial infarction, contractile dysfunction, increased mortality	[54,59,63,82,83,84]
*TCA cycle/anaplerotic reactions*	*−*	*− − −*	*=*	*− − −*	*Acetyl-CoA oxidation and TCA cycle intermediates replenishment*	*Reduced TCA cycle oxidative metabolism*	[16,17,18,24,25,40,44,45,50,51,52,53,56,60,75,76,78,80,85,86,87,88,89]
**Alanine**	=	−	−	−	TCA cycle, anaplerotic reactions	Inflammation and ROS production	[31,54,72,74,90,91,92]
**Glutamate**	+	+	=	+	TCA cycle and anaplerotic reactions	Stroke, cardiovascular diseases	[29,72,73,93,94]
**Phenylalanine**	+	+	=	+	Glycolysis-glucose nitric oxide production, ketone bodies formation, anaplerotic reactions	Hypertension, reduced tissue perfusion, increased insulin resistance, increased protein breakdown, and hypoalbuminemia	[28,72,95]
**Tyrosine**	+	+	=	+	Glycolysis-glucose nitric oxide production, ketone bodies formation, anaplerotic reactions	Decreased synthesis of thyroid hormones, catecholamines, neurotransmitters, or serum proteins	[28,29,63,72,95,96]
*Electron Transport Chain* *Oxidative Phosphorylation* *(ETC-OXPHOS)*	*=*	*− − −*	*−*	*− − −*	*ATP production*	*Reduced ATP production*	[4,15,18,44,61,78,79,97,98,99,100,101]
**Phosphatidylcholine, Lysophosphatidylcholine, Sphingomyelin**	=	−	=	−	Membrane fluidity, contractility, cell signaling	Membrane stiffness, ROS production, nitric oxide and ATP reduction, apoptosis, inflammation, and ion channels dysregulation	[4,28,32,59,61,92,99,100,101,102]
**Serine**	=	−	=	−	Nitric oxide production	Oxidative stress, fibrosis	[31,72,73,103,104]
**Arginine**	=	−	=	−	Nitric oxide production, anaplerotic reactions	Reduced nitric oxide, hypertension	[28,31,105,106]
**Dimethylarginine, Symmetric Dimethylarginine, and N-monomethylarginine**	=	+	=	+	Nitric oxide production	Reduced nitric oxide, hypertension	[28,31,105,106]
*Gut absorption and microbiota activity*	*+/−*	*+/−*	*+/−*	*+/−*	*Nutrients absorption*	*Production of metabolites with pathological effects*	[42,43,107,108,109,110,111,112,113,114,115,116,117,118,119,120,121,122,123]
**Trimethylamine N-oxide, trimethylamine**	=	+	=	+	Phosphatidylcholine, choline, and carnitine metabolism, chaperone, osmolyte, and piezolyte	Atherosclerosis and thrombosis, renal and liver function	[108,109,110,112,124]
**Trimethylamine**	+	+	=	+	TMAO precursor endogenous and esogenous	Obesity, diabetes, cardiovascular, and renal disorders	[113,114]
**Short-chain fatty acids**	=	+	=/−	+	ATP production, ketone bodies formation, FA metabolism	Hypertension, hypertrophy, and fibrosis	[42,43,110,115,116,117]
**Bile acids**	−	=	=	=	Vascular tone and blood pressure regulation, fat absorption, cholesterol, lipid, glucose metabolism	Hypertension	[110,118,119,120,121,122,123]

## Abbreviations

Heart failure, HF; heart failure with preserved ejection fraction, HFpEF; heart failure with reduced ejection fraction, HFrEF; adenosine triphosphate, ATP; fatty acids, FA; ketone bodies, KB; amino acids, AA; tricarboxylic acid cycle, TCA cycle; nicotinamide adenine dinucleotide, NADH; flavin adenine dinucleotide, FADH2; long-chain acylcarnitines, LCACs; medium-chain acylcarnitines, MCACs; small-chain acylcarnitines, SCACs; electron transport chain complexes, ETC; oxidative phosphorylation, OXPHOS; branched-chain amino acids, BCAA; trimethylamine N-oxide, TMAO; reactive oxygen species, ROS; asymmetric dimethylarginine, ADMA; symmetric dimethylarginine, SDMA; N-monomethylarginine, NMMA; New York Heart Association Functional Classification, NYHA; American Heart Association/American College of Cardiology, ACCF/AHA.

## References

[1] Suthahar N., Meijers W.C., Ho J.E., Gansevoort R.T., Voors A.A., van der Meer P., Bakker S.J.L., Heymans S., van Empel V., Schroen B. (2018). Sex-specific associations of obesity and N-terminal pro-B-type natriuretic peptide levels in the general population. Eur. J. Heart Fail.

[2] Henkens M., Remmelzwaal S., Robinson E.L., van Ballegooijen A.J., Barandiarán Aizpurua A., Verdonschot J.A.J., Raafs A.G., Weerts J., Hazebroek M.R., Sanders-van Wijk S. (2020). Risk of bias in studies investigating novel diagnostic biomarkers for heart failure with preserved ejection fraction. A systematic review. Eur. J. Heart Fail.

[3] Zile M.R., Baicu C.F. (2013). Biomarkers of diastolic dysfunction and myocardial fibrosis: Application to heart failure with a preserved ejection fraction. J. Cardiovasc. Transl. Res..

[4] Noordali H., Loudon B.L., Frenneaux M.P., Madhani M. (2018). Cardiac metabolism-A promising therapeutic target for heart failure. Pharmacol. Ther..

[5] White P.J., McGarrah R.W., Grimsrud P.A., Tso S.C., Yang W.H., Haldeman J.M., Grenier-Larouche T., An J., Lapworth A.L., Astapova I. (2018). The BCKDH kinase and phosphatase integrate BCAA and lipid metabolism via regulation of ATP-citrate lyase. Cell Metab..

[6] Hu H., Jaskiewicz J.A., Harris R.A. (1992). Ethanol and oleate inhibition of α-ketoisovalerate and 3-hydroxyisobutyrate metabolism by isolated hepatocytes. Arch. Biochem. Biophys..

[7] Paulus W.J., Tschöpe C. (2013). A novel paradigm for heart failure with preserved ejection fraction: Comorbidities drive myocardial dysfunction and remodeling through coronary microvascular endothelial inflammation. J. Am. Coll. Cardiol..

[8] Simmonds S.J., Cuijpers I., Heymans S., Jones E.A.V. (2020). Cellular and molecular differences between HFpEF and HFrEF: A step ahead in an improved pathological understanding. Cells.

[9] Contessotto P., Orbanić D., Da Costa M., Jin C., Owens P., Chantepie S., Chinello C., Newell J., Magni F., Papy-Garcia D. (2021). Elastin-like recombinamers-based hydrogel modulates post-ischemic remodeling in a non-transmural myocardial infarction in sheep. Sci. Transl. Med..

[10] Contessotto P., Pandit A. (2021). Therapies to prevent post-infarction remodelling: From repair to regeneration. Biomaterials.

[11] Li H., Xia Y.Y., Xia C.L., Li Z., Shi Y., Li X.B., Zhang J.X. (2022). Mimicking metabolic disturbance in establishing animal models of heart failure with preserved ejection fraction. Front. Physiol..

[12] Yoon S., Eom G.H. (2019). Heart failure with preserved ejection fraction: Present status and future directions. Exp. Mol. Med..

[13] Riehle C., Bauersachs J. (2019). Small animal models of heart failure. Cardiovasc. Res..

[14] Conceição G., Heinonen I., Lourenço A.P., Duncker D.J., Falcão-Pires I. (2016). Animal models of heart failure with preserved ejection fraction. Neth. Heart J..

[15] Degens H., de Brouwer K.F., Gilde A.J., Lindhout M., Willemsen P.H., Janssen B.J., van der Vusse G.J., van Bilsen M. (2006). Cardiac fatty acid metabolism is preserved in the compensated hypertrophic rat heart. Basic Res. Cardiol..

[16] Fillmore N., Levasseur J.L., Fukushima A., Wagg C.S., Wang W., Dyck J.R.B., Lopaschuk G.D. (2018). Uncoupling of glycolysis from glucose oxidation accompanies the development of heart failure with preserved ejection fraction. Mol. Med..

[17] Gomez-Arroyo J., Mizuno S., Szczepanek K., Van Tassell B., Natarajan R., dos Remedios C.G., Drake J.I., Farkas L., Kraskauskas D., Wijesinghe D.S. (2013). Metabolic gene remodeling and mitochondrial dysfunction in failing right ventricular hypertrophy secondary to pulmonary arterial hypertension. Circ. Heart Fail.

[18] Lai L., Leone T.C., Keller M.P., Martin O.J., Broman A.T., Nigro J., Kapoor K., Koves T.R., Stevens R., Ilkayeva O.R. (2014). Energy metabolic reprogramming in the hypertrophied and early stage failing heart: A multisystems approach. Circ. Heart Fail.

[19] Mori J., Alrob O.A., Wagg C.S., Harris R.A., Lopaschuk G.D., Oudit G.Y. (2013). ANG II causes insulin resistance and induces cardiac metabolic switch and inefficiency: A critical role of PDK4. Am. J. Physiol. Heart Circ. Physiol..

[20] Mori J., Basu R., McLean B.A., Das S.K., Zhang L., Patel V.B., Wagg C.S., Kassiri Z., Lopaschuk G.D., Oudit G.Y. (2012). Agonist-induced hypertrophy and diastolic dysfunction are associated with selective reduction in glucose oxidation: A metabolic contribution to heart failure with normal ejection fraction. Circ. Heart Fail.

[21] Mori J., Zhang L., Oudit G.Y., Lopaschuk G.D. (2013). Impact of the renin-angiotensin system on cardiac energy metabolism in heart failure. J. Mol. Cell Cardiol..

[22] Pellieux C., Aasum E., Larsen T.S., Montessuit C., Papageorgiou I., Pedrazzini T., Lerch R. (2006). Overexpression of angiotensinogen in the myocardium induces downregulation of the fatty acid oxidation pathway. J. Mol. Cell Cardiol..

[23] Sack M.N., Rader T.A., Park S., Bastin J., McCune S.A., Kelly D.P. (1996). Fatty acid oxidation enzyme gene expression is downregulated in the failing heart. Circulation.

[24] Umbarawan Y., Syamsunarno M., Koitabashi N., Obinata H., Yamaguchi A., Hanaoka H., Hishiki T., Hayakawa N., Sano M., Sunaga H. (2018). Myocardial fatty acid uptake through CD36 is indispensable for sufficient bioenergetic metabolism to prevent progression of pressure overload-induced heart failure. Sci. Rep..

[25] Umbarawan Y., Syamsunarno M., Koitabashi N., Yamaguchi A., Hanaoka H., Hishiki T., Nagahata-Naito Y., Obinata H., Sano M., Sunaga H. (2018). Glucose is preferentially utilized for biomass synthesis in pressure-overloaded hearts: Evidence from fatty acid-binding protein-4 and -5 knockout mice. Cardiovasc. Res..

[26] Murashige D., Jang C., Neinast M., Edwards J.J., Cowan A., Hyman M.C., Rabinowitz J.D., Frankel D.S., Arany Z. (2020). Comprehensive quantification of fuel use by the failing and nonfailing human heart. Science.

[27] Hunter W.G., Kelly J.P., McGarrah R.W., Khouri M.G., Craig D., Haynes C., Ilkayeva O., Stevens R.D., Bain J.R., Muehlbauer M.J. (2016). Metabolomic profiling identifies novel circulating biomarkers of mitochondrial dysfunction differentially elevated in heart failure with preserved versus reduced ejection fraction: Evidence for shared metabolic impairments in clinical heart failure. J. Am. Heart Assoc..

[28] Zhao H., Shui B., Zhao Q., Hu Z., Shu Q., Su M., Zhang Y., Ni Y. (2021). Quantitative metabolomics reveals heart failure with midrange ejection fraction as a distinct phenotype of heart failure. Can. J. Cardiol..

[29] Cheng M.L., Wang C.H., Shiao M.S., Liu M.H., Huang Y.Y., Huang C.Y., Mao C.T., Lin J.F., Ho H.Y., Yang N.I. (2015). Metabolic disturbances identified in plasma are associated with outcomes in patients with heart failure: Diagnostic and prognostic value of metabolomics. J. Am. Coll. Cardiol..

[30] Chen W.S., Liu M.H., Cheng M.L., Wang C.H. (2020). Decreases in circulating concentrations of short-chain acylcarnitines are associated with systolic function improvement after decompensated heart failure. Int. Heart J..

[31] Hage C., Löfgren L., Michopoulos F., Nilsson R., Davidsson P., Kumar C., Ekström M., Eriksson M.J., Lyngå P., Persson B. (2020). Metabolomic profile in HFpEF vs HFrEF patients. J. Card. Fail.

[32] Zordoky B.N., Sung M.M., Ezekowitz J., Mandal R., Han B., Bjorndahl T.C., Bouatra S., Anderson T., Oudit G.Y., Wishart D.S. (2015). Metabolomic fingerprint of heart failure with preserved ejection fraction. PLoS ONE.

[33] Wang H., Anstrom K., Ilkayeva O., Muehlbauer M.J., Bain J.R., McNulty S., Newgard C.B., Kraus W.E., Hernandez A., Felker G.M. (2017). Sildenafil treatment in heart failure with preserved ejection fraction: Targeted metabolomic profiling in the relax trial. JAMA Cardiol..

[34] Tourki B., Kain V., Shaikh S.R., Leroy X., Serhan C.N., Halade G.V. (2020). Deficit of resolution receptor magnifies inflammatory leukocyte directed cardiorenal and endothelial dysfunction with signs of cardiomyopathy of obesity. FASEB J..

[35] Ferro F., Ouillé A., Tran T.A., Fontanaud P., Bois P., Babuty D., Labarthe F., Le Guennec J.Y. (2012). Long-chain acylcarnitines regulate the hERG channel. PLoS ONE.

[36] Aguer C., McCoin C.S., Knotts T.A., Thrush A.B., Ono-Moore K., McPherson R., Dent R., Hwang D.H., Adams S.H., Harper M.E. (2015). Acylcarnitines: Potential implications for skeletal muscle insulin resistance. FASEB J..

[37] Ahmad T., Kelly J.P., McGarrah R.W., Hellkamp A.S., Fiuzat M., Testani J.M., Wang T.S., Verma A., Samsky M.D., Donahue M.P. (2016). Prognostic implications of long-chain acylcarnitines in heart failure and reversibility with mechanical circulatory support. J. Am. Coll. Cardiol..

[38] Ruiz M., Labarthe F., Fortier A., Bouchard B., Thompson Legault J., Bolduc V., Rigal O., Chen J., Ducharme A., Crawford P.A. (2017). Circulating acylcarnitine profile in human heart failure: A surrogate of fatty acid metabolic dysregulation in mitochondria and beyond. Am. J. Physiol. Heart Circ. Physiol..

[39] Aitken-Buck H.M., Krause J., Zeller T., Jones P.P., Lamberts R.R. (2020). Long-chain acylcarnitines and cardiac excitation-contraction coupling: Links to arrhythmias. Front. Physiol..

[40] Albert C.L., Tang W.H.W. (2018). Metabolic biomarkers in heart failure. Heart Fail Clin..

[41] Stride N., Larsen S., Hey-Mogensen M., Sander K., Lund J.T., Gustafsson F., Køber L., Dela F. (2013). Decreased mitochondrial oxidative phosphorylation capacity in the human heart with left ventricular systolic dysfunction. Eur. J. Heart Fail.

[42] Chen X.F., Chen X., Tang X. (2020). Short-chain fatty acid, acylation and cardiovascular diseases. Clin. Sci..

[43] Carley A.N., Maurya S.K., Fasano M., Wang Y., Selzman C.H., Drakos S.G., Lewandowski E.D. (2021). Short-chain fatty acids outpace ketone oxidation in the failing heart. Circulation.

[44] Aubert G., Martin O.J., Horton J.L., Lai L., Vega R.B., Leone T.C., Koves T., Gardell S.J., Krüger M., Hoppel C.L. (2016). The failing heart relies on ketone bodies as a fuel. Circulation.

[45] Horton J.L., Davidson M.T., Kurishima C., Vega R.B., Powers J.C., Matsuura T.R., Petucci C., Lewandowski E.D., Crawford P.A., Muoio D.M. (2019). The failing heart utilizes 3-hydroxybutyrate as a metabolic stress defense. JCI Insight.

[46] Karwi Q.G., Zhang L., Wagg C.S., Wang W., Ghandi M., Thai D., Yan H., Ussher J.R., Oudit G.Y., Lopaschuk G.D. (2019). Targeting the glucagon receptor improves cardiac function and enhances insulin sensitivity following a myocardial infarction. Cardiovasc. Diabetol..

[47] Lopaschuk G.D., Karwi Q.G., Tian R., Wende A.R., Abel E.D. (2021). Cardiac energy metabolism in heart failure. Circ. Res..

[48] Schugar R.C., Moll A.R., André d’Avignon D., Weinheimer C.J., Kovacs A., Crawford P.A. (2014). Cardiomyocyte-specific deficiency of ketone body metabolism promotes accelerated pathological remodeling. Mol. Metab..

[49] Shimazu T., Hirschey M.D., Newman J., He W., Shirakawa K., Le Moan N., Grueter C.A., Lim H., Saunders L.R., Stevens R.D. (2013). Suppression of oxidative stress by β-hydroxybutyrate, an endogenous histone deacetylase inhibitor. Science.

[50] Youm Y.H., Nguyen K.Y., Grant R.W., Goldberg E.L., Bodogai M., Kim D., D’Agostino D., Planavsky N., Lupfer C., Kanneganti T.D. (2015). The ketone metabolite β-hydroxybutyrate blocks NLRP3 inflammasome-mediated inflammatory disease. Nat. Med..

[51] De Jong K.A., Lopaschuk G.D. (2017). Complex energy metabolic changes in heart failure with preserved ejection fraction and heart failure with reduced ejection fraction. Can. J. Cardiol..

[52] Deng Y., Xie M., Li Q., Xu X., Ou W., Zhang Y., Xiao H., Yu H., Zheng Y., Liang Y. (2021). Targeting mitochondria-inflammation circuit by β-hydroxybutyrate mitigates HFpEF. Circ. Res..

[53] Bedi K.C., Snyder N.W., Brandimarto J., Aziz M., Mesaros C., Worth A.J., Wang L.L., Javaheri A., Blair I.A., Margulies K.B. (2016). Evidence for intramyocardial disruption of lipid metabolism and increased myocardial ketone utilization in advanced human heart failure. Circulation.

[54] Du Z., Shen A., Huang Y., Su L., Lai W., Wang P., Xie Z., Xie Z., Zeng Q., Ren H. (2014). 1H-NMR-based metabolic analysis of human serum reveals novel markers of myocardial energy expenditure in heart failure patients. PLoS ONE.

[55] Flores-Guerrero J.L., Westenbrink B.D., Connelly M.A., Otvos J.D., Groothof D., Shalaurova I., Garcia E., Navis G., de Boer R.A., Bakker S.J.L. (2021). Association of beta-hydroxybutyrate with development of heart failure: Sex differences in a Dutch population cohort. Eur J. Clin. Investig..

[56] Lommi J., Kupari M., Koskinen P., Näveri H., Leinonen H., Pulkki K., Härkönen M. (1996). Blood ketone bodies in congestive heart failure. J. Am. Coll. Cardiol..

[57] Voros G., Ector J., Garweg C., Droogne W., Van Cleemput J., Peersman N., Vermeersch P., Janssens S. (2018). Increased cardiac uptake of ketone bodies and free fatty acids in human heart failure and hypertrophic left ventricular remodeling. Circ. Heart Fail.

[58] Yokokawa T., Yoshihisa A., Kanno Y., Abe S., Misaka T., Yamada S., Kaneshiro T., Sato T., Oikawa M., Kobayashi A. (2019). Circulating acetoacetate is associated with poor prognosis in heart failure patients. Int. J. Cardiol. Heart Vasc..

[59] Andersson C., Liu C., Cheng S., Wang T.J., Gerszten R.E., Larson M.G., Vasan R.S. (2020). Metabolomic signatures of cardiac remodelling and heart failure risk in the community. ESC Heart Fail.

[60] Lommi J., Koskinen P., Näveri H., Härkönen M., Kupari M. (1997). Heart failure ketosis. J. Intern. Med..

[61] Puchalska P., Crawford P.A. (2017). Multi-dimensional roles of ketone bodies in fuel metabolism, signaling, and therapeutics. Cell Metab..

[62] Chouchani E.T., Pell V.R., Gaude E., Aksentijević D., Sundier S.Y., Robb E.L., Logan A., Nadtochiy S.M., Ord E.N.J., Smith A.C. (2014). Ischaemic accumulation of succinate controls reperfusion injury through mitochondrial ROS. Nature.

[63] Stryeck S., Gastrager M., Degoricija V., Trbušić M., Potočnjak I., Radulović B., Pregartner G., Berghold A., Madl T., Frank S. (2019). Serum concentrations of citrate, tyrosine, 2- and 3- hydroxybutyrate are associated with increased 3-month mortality in acute heart failure patients. Sci Rep..

[64] Tannahill G.M., Curtis A.M., Adamik J., Palsson-McDermott E.M., McGettrick A.F., Goel G., Frezza C., Bernard N.J., Kelly B., Foley N.H. (2013). Succinate is an inflammatory signal that induces IL-1β through HIF-1α. Nature.

[65] Fillmore N., Wagg C.S., Zhang L., Fukushima A., Lopaschuk G.D. (2018). Cardiac branched-chain amino acid oxidation is reduced during insulin resistance in the heart. Am. J. Physiol. Endocrinol. Metab..

[66] Li R., He H., Fang S., Hua Y., Yang X., Yuan Y., Liang S., Liu P., Tian Y., Xu F. (2019). Time series characteristics of serum branched-chain amino acids for early diagnosis of chronic heart failure. J. Proteome Res..

[67] Sansbury B.E., DeMartino A.M., Xie Z., Brooks A.C., Brainard R.E., Watson L.J., DeFilippis A.P., Cummins T.D., Harbeson M.A., Brittian K.R. (2014). Metabolomic analysis of pressure-overloaded and infarcted mouse hearts. Circ. Heart Fail.

[68] Sun H., Olson K.C., Gao C., Prosdocimo D.A., Zhou M., Wang Z., Jeyaraj D., Youn J.Y., Ren S., Liu Y. (2016). Catabolic defect of branched-chain amino acids promotes heart failure. Circulation.

[69] Guo N., Yang D., Wang X., Dai J., Wang M., Lei Y. (2014). Metabonomic study of chronic heart failure and effects of chinese herbal decoction in rats. J. Chromatogr. A.

[70] Jang C., Oh S.F., Wada S., Rowe G.C., Liu L., Chan M.C., Rhee J., Hoshino A., Kim B., Ibrahim A. (2016). A branched-chain amino acid metabolite drives vascular fatty acid transport and causes insulin resistance. Nat. Med..

[71] Kadota Y., Toyoda T., Hayashi-Kato M., Kitaura Y., Shimomura Y. (2015). Octanoic acid promotes branched-chain amino acid catabolisms via the inhibition of hepatic branched-chain alpha-keto acid dehydrogenase kinase in rats. Metabolism.

[72] Saleem T.H., Algowhary M., Kamel F.E.M., El-Mahdy R.I. (2020). Plasma amino acid metabolomic pattern in heart failure patients with either preserved or reduced ejection fraction: The relation to established risk variables and prognosis. Biomed. Chromatogr..

[73] Wang C.H., Cheng M.L., Liu M.H. (2018). Amino acid-based metabolic panel provides robust prognostic value additive to b-natriuretic peptide and traditional risk factors in heart failure. Dis. Markers.

[74] Deidda M., Piras C., Cadeddu Dessalvi C., Congia D., Locci E., Ascedu F., De Candia G., Cadeddu M., Lai G., Pirisi R. (2017). Blood metabolomic fingerprint is distinct in healthy coronary and in stenosing or microvascular ischemic heart disease. J. Transl. Med..

[75] Chandler M.P., Kerner J., Huang H., Vazquez E., Reszko A., Martini W.Z., Hoppel C.L., Imai M., Rastogi S., Sabbah H.N. (2004). Moderate severity heart failure does not involve a downregulation of myocardial fatty acid oxidation. Am. J. Physiol. Heart Circ. Physiol..

[76] Turer A., Altamirano F., Schiattarella G.G., May H., Gillette T.G., Malloy C.R., Merritt M.E. (2019). Remodeling of substrate consumption in the murine sTAC model of heart failure. J. Mol. Cell. Cardiol..

[77] Zeng H., Chen J.X. (2019). Sirtuin 3, endothelial metabolic reprogramming, and heart failure with preserved ejection fraction. J. Cardiovasc. Pharmacol..

[78] Zhang L., Jaswal J.S., Ussher J.R., Sankaralingam S., Wagg C., Zaugg M., Lopaschuk G.D. (2013). Cardiac insulin-resistance and decreased mitochondrial energy production precede the development of systolic heart failure after pressure-overload hypertrophy. Circ. Heart Fail.

[79] Fernandez-Caggiano M., Eaton P. (2021). Heart failure-emerging roles for the mitochondrial pyruvate carrier. Cell Death Differ..

[80] Hue L., Taegtmeyer H. (2009). The Randle cycle revisited: A new head for an old hat. Am. J. Physiol. Endocrinol. Metab..

[81] Morimoto S., Goto T. (2000). Role of troponin I isoform switching in determining the pH sensitivity of Ca^2+^ regulation in developing rabbit cardiac muscle. Biochem. Biophys. Res. Commun..

[82] Contaifer D., Buckley L.F., Wohlford G., Kumar N.G., Morriss J.M., Ranasinghe A.D., Carbone S., Canada J.M., Trankle C., Abbate A. (2019). Metabolic modulation predicts heart failure tests performance. PLoS ONE.

[83] Desmoulin F., Galinier M., Trouillet C., Berry M., Delmas C., Turkieh A., Massabuau P., Taegtmeyer H., Smih F., Rouet P. (2013). Metabonomics analysis of plasma reveals the lactate to cholesterol ratio as an independent prognostic factor of short-term mortality in acute heart failure. PLoS ONE.

[84] Lazzeri C., Valente S., Chiostri M., Picariello C., Gensini G.F. (2012). Lactate in the acute phase of ST-elevation myocardial infarction treated with mechanical revascularization: A single-center experience. Am. J. Emerg. Med..

[85] Shibayama J., Yuzyuk T.N., Cox J., Makaju A., Miller M., Lichter J., Li H., Leavy J.D., Franklin S., Zaitsev A.V. (2015). Metabolic remodeling in moderate synchronous versus dyssynchronous pacing-induced heart failure: Integrated metabolomics and proteomics study. PLoS ONE.

[86] Sakao S., Kawakami E., Shoji H., Naito A., Miwa H., Suda R., Sanada T.J., Tanabe N., Tatsumi K. (2021). Metabolic remodeling in the right ventricle of rats with severe pulmonary arterial hypertension. Mol. Med. Rep..

[87] Forsey R.G., Reid K., Brosnan J.T. (1987). Competition between fatty acids and carbohydrate or ketone bodies as metabolic fuels for the isolated perfused heart. Can. J. Physiol. Pharmacol..

[88] Purmal C., Kucejova B., Sherry A.D., Burgess S.C., Malloy C.R., Merritt M.E. (2014). Propionate stimulates pyruvate oxidation in the presence of acetate. Am. J. Physiol. Heart Circ. Physiol..

[89] Russell R.R., Taegtmeyer H. (1991). Changes in citric acid cycle flux and anaplerosis antedate the functional decline in isolated rat hearts utilizing acetoacetate. J. Clin. Investig..

[90] Hunter W.G., Kelly J.P., McGarrah R.W., Kraus W.E., Shah S.H. (2016). Metabolic dysfunction in heart failure: Diagnostic, prognostic, and pathophysiologic insights from metabolomic profiling. Curr. Heart Fail Rep..

[91] Parker S.J., Amendola C.R., Hollinshead K.E.R., Yu Q., Yamamoto K., Encarnación-Rosado J., Rose R.E., LaRue M.M., Sohn A.S.W., Biancur D.E. (2020). Selective alanine transporter utilization creates a targetable metabolic niche in pancreatic cancer. Cancer Discov..

[92] Zhou J., Chen X., Chen W., Zhong L., Cui M. (2021). Comprehensive plasma metabolomic and lipidomic analyses reveal potential biomarkers for heart failure. Mol. Cell. Biochem..

[93] Arsenian M. (1998). Potential cardiovascular applications of glutamate, aspartate, and other amino acids. Clin. Cardiol..

[94] Zheng Y., Hu F.B., Ruiz-Canela M., Clish C.B., Dennis C., Salas-Salvado J., Hruby A., Liang L., Toledo E., Corella D. (2016). Metabolites of glutamate metabolism are associated with incident cardiovascular events in the PREDIMED PREvención con Dieta MEDiterránea (PREDIMED) trial. J. Am. Heart Assoc..

[95] Hiraiwa H., Okumura T., Kondo T., Kato T., Kazama S., Kimura Y., Ishihara T., Iwata E., Shimojo M., Kondo S. (2021). Prognostic value of leucine/phenylalanine ratio as an amino acid profile of heart failure. Heart Vessel..

[96] Wang C.H., Cheng M.L., Liu M.H. (2018). Simplified plasma essential amino acid-based profiling provides metabolic information and prognostic value additive to traditional risk factors in heart failure. Amino Acids.

[97] Blain A.M., Straub V.W. (2011). δ-Sarcoglycan-deficient muscular dystrophy: From discovery to therapeutic approaches. Skelet. Muscle.

[98] Okumura K., Yamada Y., Kondo J., Hashimoto H., Ito T., Kitoh J. (1991). Decreased 1,2-diacylglycerol levels in myopathic hamster hearts during the development of heart failure. J. Mol. Cell. Cardiol..

[99] Rosca M.G., Tandler B., Hoppel C.L. (2013). Mitochondria in cardiac hypertrophy and heart failure. J. Mol. Cell. Cardiol..

[100] Szeto H.H. (2014). First-in-class cardiolipin-protective compound as a therapeutic agent to restore mitochondrial bioenergetics. Br. J. Pharmacol..

[101] Wende A.R., Brahma M.K., McGinnis G.R., Young M.E. (2017). Metabolic origins of heart failure. JACC Basic Transl. Sci..

[102] Basu Ball W., Neff J.K., Gohil V.M. (2018). The role of nonbilayer phospholipids in mitochondrial structure and function. FEBS Lett..

[103] Padrón-Barthe L., Villalba-Orero M., Gómez-Salinero J.M., Acín-Pérez R., Cogliati S., López-Olañeta M., Ortiz-Sánchez P., Bonzón-Kulichenko E., Vázquez J., García-Pavía P. (2018). Activation of serine one-carbon metabolism by calcineurin Aβ1 reduces myocardial hypertrophy and improves ventricular function. J. Am. Coll. Cardiol..

[104] Zhou X., He L., Zuo S., Zhang Y., Wan D., Long C., Huang P., Wu X., Wu C., Liu G. (2018). Serine prevented high-fat diet-induced oxidative stress by activating AMPK and epigenetically modulating the expression of glutathione synthesis-related genes. Biochim. Biophys. Acta Mol. Basis Dis..

[105] Shao Z., Wang Z., Shrestha K., Thakur A., Borowski A.G., Sweet W., Thomas J.D., Moravec C.S., Hazen S.L., Tang W.H. (2012). Pulmonary hypertension associated with advanced systolic heart failure: Dysregulated arginine metabolism and importance of compensatory dimethylarginine dimethylaminohydrolase-1. J. Am. Coll. Cardiol..

[106] Tang W.H., Tong W., Shrestha K., Wang Z., Levison B.S., Delfraino B., Hu B., Troughton R.W., Klein A.L., Hazen S.L. (2008). Differential effects of arginine methylation on diastolic dysfunction and disease progression in patients with chronic systolic heart failure. Eur. Heart J..

[107] O’Donovan A.N., Herisson F.M., Fouhy F., Ryan P.M., Whelan D., Johnson C.N., Cluzel G., Ross R.P., Stanton C., Caplice N.M. (2020). Gut microbiome of a porcine model of metabolic syndrome and HF-pEF. Am. J. Physiol. Heart Circ. Physiol..

[108] Guo F., Qiu X., Tan Z., Li Z., Ouyang D. (2020). Plasma trimethylamine n-oxide is associated with renal function in patients with heart failure with preserved ejection fraction. BMC Cardiovasc. Disord..

[109] Schuett K., Kleber M.E., Scharnagl H., Lorkowski S., März W., Niessner A., Marx N., Meinitzer A. (2017). Trimethylamine-N-oxide and heart failure with reduced versus preserved ejection fraction. J. Am. Coll. Cardiol..

[110] Tang W.H.W., Li D.Y., Hazen S.L. (2019). Dietary metabolism, the gut microbiome, and heart failure. Nat. Rev. Cardiol..

[111] Zhang Y., Wang Y., Ke B., Du J. (2021). TMAO: How gut microbiota contributes to heart failure. Transl. Res..

[112] Naghipour S., Cox A.J., Peart J.N., Du Toit E.F., Headrick J.P. (2021). Trimethylamine N-oxide: Heart of the microbiota-CVD nexus?. Nutr. Res. Rev..

[113] Chhibber-Goel J., Gaur A., Singhal V., Parakh N., Bhargava B., Sharma A. (2016). The complex metabolism of trimethylamine in humans: Endogenous and exogenous sources. Expert Rev. Mol. Med..

[114] Jaworska K., Hering D., Mosieniak G., Bielak-Zmijewska A., Pilz M., Konwerski M.M., Gasecka A., Kaplon-Cieslicka A., Filipiak K., Sikora E. (2019). TMA, A forgotten uremic toxin, but not TMAO, is involved in cardiovascular pathology. Toxins Basel.

[115] Yang T., Santisteban M.M., Rodriguez V., Li E., Ahmari N., Carvajal J.M., Zadeh M., Gong M., Qi Y., Zubcevic J. (2015). Gut dysbiosis is linked to hypertension. Hypertension.

[116] Pakhomov N., Baugh J.A. (2021). The role of diet-derived short-chain fatty acids in regulating cardiac pressure overload. Am. J. Physiol. Heart Circ. Physiol..

[117] Beale A.L., O’Donnell J.A., Nakai M.E., Nanayakkara S., Vizi D., Carter K., Dean E., Ribeiro R.V., Yiallourou S., Carrington M.J. (2021). The gut microbiome of heart failure with preserved ejection fraction. J. Am. Heart Assoc..

[118] Raufman J.P., Chen Y., Zimniak P., Cheng K. (2002). Deoxycholic acid conjugates are muscarinic cholinergic receptor antagonists. Pharmacology.

[119] Wang H., Chen J., Hollister K., Sowers L.C., Forman B.M. (1999). Endogenous bile acids are ligands for the nuclear receptor FXR/BAR. Mol. Cell.

[120] Ljubuncic P., Said O., Ehrlich Y., Meddings J.B., Shaffer E.A., Bomzon A. (2000). On the in vitro vasoactivity of bile acids. Br. J. Pharmacol..

[121] Mayerhofer C.C.K., Ueland T., Broch K., Vincent R.P., Cross G.F., Dahl C.P., Aukrust P., Gullestad L., Hov J.R., Trøseid M. (2017). Increased secondary/primary bile acid ratio in chronic heart failure. J. Card. Fail..

[122] Eblimit Z., Thevananther S., Karpen S.J., Taegtmeyer H., Moore D.D., Adorini L., Penny D.J., Desai M.S. (2018). TGR5 activation induces cytoprotective changes in the heart and improves myocardial adaptability to physiologic, inotropic, and pressure-induced stress in mice. Cardiovasc. Ther..

[123] von Haehling S., Schefold J.C., Jankowska E.A., Springer J., Vazir A., Kalra P.R., Sandek A., Fauler G., Stojakovic T., Trauner M. (2012). Ursodeoxycholic acid in patients with chronic heart failure: A double-blind, randomized, placebo-controlled, crossover trial. J. Am. Coll. Cardiol..

[124] Zhang J., Wang L., Cai J., Lei A., Liu C., Lin R., Jia L., Fu Y. (2021). Gut microbial metabolite TMAO portends prognosis in acute ischemic stroke. J. Neuroimmunol..

[125] Cotter D.G., Schugar R.C., Crawford P.A. (2013). Ketone body metabolism and cardiovascular disease. Am. J. Physiol. Heart Circ. Physiol..

[126] Berg J.M., Tymoczko J.L., Stryer L. (2007). Protein turnover and amino acid catabolism. Biochemistry.

[127] Shao D., Villet O., Zhang Z., Choi S.W., Yan J., Ritterhoff J., Gu H., Djukovic D., Christodoulou D., Kolwicz S.C. (2018). Glucose promotes cell growth by suppressing branched-chain amino acid degradation. Nat. Commun..

[128] Wang J., Li Z., Chen J., Zhao H., Luo L., Chen C., Xu X., Zhang W., Gao K., Li B. (2013). Metabolomic identification of diagnostic plasma biomarkers in humans with chronic heart failure. Mol. Biosyst..

[129] Tsuji S., Koyama S., Taniguchi R., Fujiwara T., Fujiwara H., Sato Y. (2018). Nutritional status of outpatients with chronic stable heart failure based on serum amino acid concentration. J. Cardiol..

[130] Zhenyukh O., González-Amor M., Rodrigues-Diez R.R., Esteban V., Ruiz-Ortega M., Salaices M., Mas S., Briones A.M., Egido J. (2018). Branched-chain amino acids promote endothelial dysfunction through increased reactive oxygen species generation and inflammation. J. Cell. Mol. Med..

[131] Ussher J.R., Jaswal J.S., Lopaschuk G.D. (2012). Pyridine nucleotide regulation of cardiac intermediary metabolism. Circ. Res..

[132] Martínez-Reyes I., Chandel N.S. (2020). Mitochondrial TCA cycle metabolites control physiology and disease. Nat. Commun..

[133] Wang C.H., Cheng M.L., Liu M.H., Fu T.C. (2019). Amino acid-based metabolic profile provides functional assessment and prognostic value for heart failure outpatients. Dis. Markers.

[134] Neubauer S. (2007). The failing heart-an engine out of fuel. N. Engl. J. Med..

[135] Schwarz K., Siddiqi N., Singh S., Neil C.J., Dawson D.K., Frenneaux M.P. (2014). The breathing heart-mitochondrial respiratory chain dysfunction in cardiac disease. Int. J. Cardiol..

[136] Brown D.A., Perry J.B., Allen M.E., Sabbah H.N., Stauffer B.L., Shaikh S.R., Cleland J.G., Colucci W.S., Butler J., Voors A.A. (2017). Expert consensus document: Mitochondrial function as a therapeutic target in heart failure. Nat. Rev. Cardiol..

[137] Stenemo M., Ganna A., Salihovic S., Nowak C., Sundström J., Giedraitis V., Broeckling C.D., Prenni J.E., Svensson P., Magnusson P.K.E. (2019). The metabolites urobilin and sphingomyelin (30:1) are associated with incident heart failure in the general population. ESC Heart Fail..

[138] Lee D.S., Gona P., Vasan R.S., Larson M.G., Benjamin E.J., Wang T.J., Tu J.V., Levy D. (2009). Relation of disease pathogenesis and risk factors to heart failure with preserved or reduced ejection fraction: Insights from the framingham heart study of the national heart, lung, and blood institute. Circulation.

[139] Savji N., Meijers W.C., Bartz T.M., Bhambhani V., Cushman M., Nayor M., Kizer J.R., Sarma A., Blaha M.J., Gansevoort R.T. (2018). The association of obesity and cardiometabolic traits with incident HFpEF and HFrEF. JACC Heart Fail..

[140] Li H., Hastings M.H., Rhee J., Trager L.E., Roh J.D., Rosenzweig A. (2020). Targeting age-related pathways in heart failure. Circ. Res..

[141] Sandesara P.B., O’Neal W.T., Kelli H.M., Samman-Tahhan A., Hammadah M., Quyyumi A.A., Sperling L.S. (2018). The prognostic significance of diabetes and microvascular complications in patients with heart failure with preserved ejection fraction. Diabetes Care.

[142] Tomasoni D., Adamo M., Anker M.S., von Haehling S., Coats A.J.S., Metra M. (2020). Heart failure in the last year: Progress and perspective. ESC Heart Fail..

[143] Sabbatini A.R., Kararigas G. (2020). Menopause-related estrogen decrease and the pathogenesis of HFpEF: JACC review topic of the week. J. Am. Coll. Cardiol..

[144] Lam C.S.P., Arnott C., Beale A.L., Chandramouli C., Hilfiker-Kleiner D., Kaye D.M., Ky B., Santema B.T., Sliwa K., Voors A.A. (2019). Sex differences in heart failure. Eur. Heart J..

[145] Ashrafian H., Sounderajah V., Glen R., Ebbels T., Blaise B.J., Kalra D., Kultima K., Spjuth O., Tenori L., Salek R.M. (2021). Metabolomics: The stethoscope for the twenty-first century. Med. Princ. Pract..

